# Liver fluke infections by *Amphimerus* sp. (Digenea: Opisthorchiidae) in definitive and fish intermediate hosts in Manabí province, Ecuador

**DOI:** 10.1371/journal.pntd.0008286

**Published:** 2020-06-29

**Authors:** Daniel Romero-Alvarez, Gabriela Valverde-Muñoz, Manuel Calvopina, Maira Rojas, William Cevallos, Hideo Kumazawa, Hidekazu Takagi, Hiromu Sugiyama

**Affiliations:** 1 Department of Ecology & Evolutionary Biology and Biodiversity Institute, University of Kansas, Lawrence, Kansas, United States of America; 2 OneHealth Research Group-Facultad de Ciencias de la Salud, Universidad de las Américas, Quito, Ecuador; 3 English for Academic Purposes, Johnson County Community College, Lawrence, Kansas, United States of America; 4 Facultad de Ciencias de la Salud, Universidad de las Américas, Quito, Ecuador; 5 Instituto de Biomedicina, Universidad Central del Ecuador, Quito, Ecuador; 6 Department of Parasitology, Kochi Medical School, Nankoku, Japan; 7 Department of Microbiology and Immunology, Aichi Medical University School of Medicine, Aichi, Japan; 8 Department of Parasitology, National Institute of Infectious Diseases, Tokyo, Japan; Seoul National University College of Medicine, REPUBLIC OF KOREA

## Abstract

*Amphimerus* sp. is a fluke that dwells in the biliary tracts of vertebrate definitive hosts including humans, domestic, and wild mammals in Latin America. Opisthorchiid liver infections are rarely studied in the Americas confirming its status as a neglected tropical disease. In Ecuador, small trematode eggs were reported in human cases from the province of Manabí in 1949, and recently, *Amphimerus* sp. adults were recovered from human and reservoir hosts in the province of Esmeraldas. Due to the lack of research on the infectious sources of *Amphimerus* sp. in the continent, we have developed a series of epidemiological studies with parasitological and molecular techniques to elucidate the endemicity of opisthorchiid fluke infections. We developed a cross-sectional study in three communities at Pedro Pablo Gómez parish in the province of Manabí, Ecuador. We examined a total of 176 fecal samples to detect opisthorchiid eggs, and four fish species to find opisthorchiid metacercariae. To study adult worms, we treated and purged seven patients in a family and dissected the livers of a dog and a cat infected. We observed morphological features of adults and metacercariae and used polymerase chain reaction with restricted fragment length polymorphism (PCR-RFLP) and DNA sequencing of a section of the ITS2 gene for identification. Small trematode eggs were detected in 63 (35.8%) out of 176 fecal samples of residents in the three study sites. Adult opisthorchiid flukes were recovered from human patients, a dog and a cat, and they were morphologically and molecularly identified as *Amphimerus* sp. Opisthorchiid metacercariae were also identified molecularly as *Amphimerus* sp. in four fish species, i.e., *Rhoadsia altipinna*, *Bryconamericus bucay*, *Andinoacara rivulatus*, and *Piabucina aureoguttata*. Metacercariae of the heterophyid *Haplorchis pumilio* were also found in the four fish species examined. This is the first study to confirm the current endemicity of *Amphimerus* sp. in Pedro Pablo Gómez, Manabí, Ecuador. The adult worms isolated here shared morphological characteristics with previous *Amphimerus* sp. descriptions and were molecularly similar to *Amphimerus* sp. described in the province of Esmeraldas. Moreover, this study is the first to document four fish species as infection sources of *Amphimerus* sp. detected via a molecular protocol targeting the metacercariae of the parasite. Fish species identified here should be targeted for public health campaigns to avoid further human liver-fluke infections by *Amphimerus* sp. or potential intestinal-fluke infections by *H*. *pumilio* or others.

## Author summary

Among neglected tropical diseases, foodborne trematodiases rank among the least studied, especially in the Americas. In Ecuador, a parasite of the genus *Amphimerus*, family Opisthorchiidae, has been found in the biliary ducts of humans, cats, and dogs, in the northern province of Esmeraldas. Reports as old as 1949 document opisthorchiid infections in the coastal province of Manabí. Given a lack of studies elucidating the infective larval stage of these flukes in Ecuador, we decided to conduct parasitological and molecular experiments to characterize the presence of these human liver infections in the area. We found adult *Amphimerus* sp. flukes in humans and other domestic animals, and its metacercariae in four edible freshwater fish species: *Rhoadsia altipinna*, *Bryconamericus bucay*, *Andinoacara rivulatus*, and *Piabucina aureoguttata*. Moreover, we found a prevalence of 35.8% of small trematode eggs in humans. Finally, we also found the metacercariae of the intestinal fluke *Haplorchis pumilio* in all fish species examined. Our findings confirm the area of Pedro Pablo Gómez as an endemic region for opisthorchiid liver infections due to *Amphimerus* sp., and expand the human differential diagnosis of small trematode eggs to liver and intestinal flukes. Consumption of undercooked freshwater fish of the species identified here could lead to *Amphimerus* sp. infections. Surveillance in other areas known for raw fish consumption would likely unveil new cases of foodborne trematodiases in Ecuador and other regions.

## Introduction

Foodborne trematodiases are an array of zoonotic parasitic infections caused by non-segmented flatworms (phylum Platyhelminthes) that are categorized by the World Health Organization (WHO) among the most neglected tropical diseases [1–3]. Approximately 56 million people in the world are affected by one of the following trematodes: *Clonorchis sinensis*, *Opisthorchis viverrini*, *O*. *felineus*, and *Fasciola* spp., causing liver fluke infections; *Haplorchis* spp., *Metagonimus* spp., *Echinostoma* spp., and others, causing intestinal infections; and *Paragonimus* spp., causing lung fluke infections [4,5]. Among these, the trematodes of the family Opisthorchiidae, *Clonorchis sinensis* and *Opisthorchis* spp., affect more than 24 million people, especially in Asian countries [6]; some have been demonstrated as carcinogenic agents involved in the etiology of cholangiocarcinoma in humans [7,8].

Another opisthorchiid trematodes, i.e., *Amphimerus noverca*, *Metorchis conjunctus*, *M*. *bilis*, and *Pseudamphistomum truncatum*, have also been reported as human parasites [5,9]. However, autochthonous human opisthorchiid infections in the Americas are understudied [5,9,10]. In South America, opisthorchiid liver flukes identified in humans were reported for the first time in 1949 in communities of Manabí, a coastal province of Ecuador. Rodríguez et al. (1949) documented trematode eggs in humans and dogs, recovered adult flukes from dogs, and, based on their morphological characteristics, designated the parasite species as *Opisthorchis guayaquilensis* [11]. In 2008, Moreira et al. reported small trematode eggs in riverine communities of the Cayapas River at the Esmeraldas province of the same country, suggesting the presence of *O*. *guayaquilensis* [12]. An in-depth investigation of the parasite in the same province, based on scanning electron microscopy of eggs and morphological characteristics of adult flukes recovered from the biliary ducts of humans, dogs, and cats, identified the parasite as *Amphimerus* sp. [13,14].

Parasitological diagnosis of opisthorchiid trematodes affecting biliary ducts is usually achieved by examining stools via different techniques such as direct smear, simple sedimentation in tube, Kato-Katz, or formalin-ether concentration [5,15,16]. When present, pyriform operculated eggs can be identified through simple microscopy; however, these characteristics are also shared by eggs from the intestinal flukes of the family Heterophyidae (e.g., *Haplorchis* spp. or *Metagonimus* spp.). Thus, when these eggs are found, they are identified only as ‘small trematode eggs’ [17–19]. Molecular and immunological diagnostic tools have been developed to discriminate between opisthorchiid and heterophyid eggs in Asia where their burden has been studied extensively (e.g., Thailand) [10,20]. In Ecuador, *Amphimerus* sp. diagnosis has been explored using multiple coproparasitological methods, which to today remain the gold standard for small trematode egg identification [15]. Recently, immunological and molecular methods based on enzyme-linked immunosorbent assay (ELISA) and loop-mediated isothermal amplification (LAMP) have been developed, which could improve case diagnosis and detection [21–23].

Opisthorchiid liver flukes have complex life cycles, including multiple larval forms and two intermediate hosts [5,10,24,25]. Briefly, eggs arriving into freshwater water sources (e.g., streams, lakes, ponds) are ingested by prosobranch molluscs, the first intermediate hosts, in which miracidia hatch and migrate along the digestive tract. Here, miracidia continue its development to form sporocysts and rediae as the first and second intra-molluscan stages respectively. Rediae produce cercariae, which escape the snail and search for fish, the second intermediate host, where they mature to become metacercariae, the infective stage. The vertebrate definitive host (e.g., mammals) ingests the metacercariae in fish bodies, and the larva inside the metacercarial cyst wall escapes and migrates from the intestines to the biliary ducts where it matures to become adult parasite and starts laying eggs [5,10,24,26].

Although small trematode eggs in humans were reported in the province of Manabí, Ecuador, with a prevalence of 7.3% (18/245 individuals) [11], follow-up studies in the area were discontinued. Fish incriminated as second intermediate hosts and details on the infective larval stage of *Amphimerus* sp. transmission are lacking even in the well-known focus of *Amphimerus* sp. in Esmeraldas province [14]. For the present report, we visited the Manabí area and collected human fecal samples and fish to (1) look for small trematode eggs in humans, (2) identify fish species involved in *Amphimerus* sp. transmission in Ecuador, and (3) gain morphological and molecular insights into its metacercariae.

## Materials and methods

### Ethics Statement

The ethics committee of the Universidad Central del Ecuador approved the project, under the license number LEC IORG 0001932, FWA 2482, IRB 2483 COBI-AMPHI-0064-11. All local residents involved in the study provided a single fecal sample voluntarily and signed a consent letter of participation in the study; underage individuals were represented by their parents or corresponding guardians. Personal data were anonymized using word and letter codes. A medical doctor treated all participants discharging parasite eggs according the guidelines of the Ministry of Public Health of Ecuador. Participants positive for small trematode eggs were treated with praziquantel, following Calvopiña et al (2011) [13]. In addition, advice regarding fish-borne parasitic diseases was offered to local residents to avoid further re-infections. Fish collection was performed under the permission of the Ministry of Environment of Ecuador (Contract MAE-DNB-CM-2018-0090). The STROBE (Strengthening the Reporting of Observational Studies in Epidemiology) checklist has been included to assure the quality on the methods and results reported in the present manuscript (See S1 File).

### Study area

**Fig 1:**
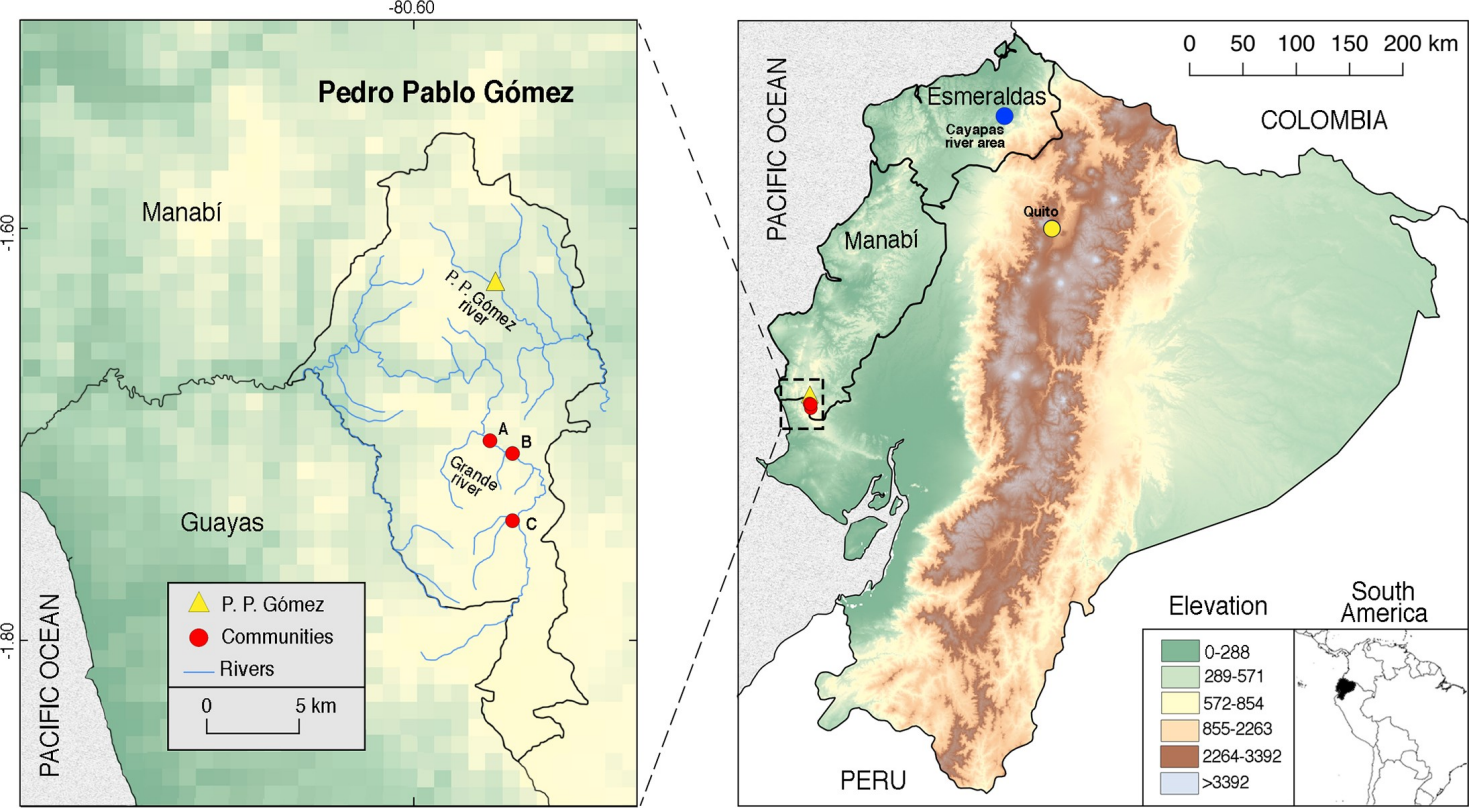
Map of the localities studied in Pedro Pablo Gómez (PPG), Manabí, Ecuador. Rural communities involved in the PPG region (left panel, red) are located at ~493 (A, latitude: -80.551933°, longitude: -1.70995°), ~483 (B, latitude: -80.562917°, longitude: -1.703733°), and ~588 (C, latitude: -80.55195°, longitude: -1.742683°) meters above sea level. *Amphimerus* sp. adult flukes were described for the first time infecting humans in the province of Esmeraldas (right panel, blue). Maps were developed using shapefiles from https://www.naturalearthdata.com/.

We developed a cross-sectional study working in three communities of Pedro Pablo Gómez (hereafter PPG), within the Jipijapa municipality, Manabí province, in the coastal region of Ecuador (Fig 1) [27]. The region belongs to the dry broadleaf forest biome [28], with dry (June to December) and rainy (January to May) seasons, mean annual temperature of 18–24°C, and annual precipitation of 500–1000 mm [27,29]. PPG has a total population of ~3500 inhabitants, with an economy based mainly on cultivation of corn, coffee, and tagua nuts. As a rural population, they depend on their own vegetable crops, livestock, hunting, and fishing as food sources; they lack purified water [27,29]. The localities involved in the study include three rural communities located at ~10.05 (A), ~10.27 (B), and ~14.64 km (C) respectively from PPG; all connected by different branches and streams of the Pedro Pablo Gómez and Grande Rivers, some with permanent water throughout the year (Fig 1) [27,29].

### Fecal examination

In each of the three communities, we met with the local people, explained the study goals, and obtained written agreement for participation by the leaders during August 2017. One fecal sample was collected from each local resident above one year old without other restriction criteria. We delivered a plastic flask filled with 70% ethanol labeled with their names, age, gender, and community. We collected samples during the next three days by visiting participants house by house. Flasks were transported to the Centro de Biomedicina at the Universidad Central del Ecuador. For coproparasitological diagnosis, we used the formalin-ether concentration method (Ritchie’s technique), as recently described [15]. In brief, stools were processed in a cycle of homogenization and dilution with 10% formalin, decantation, and centrifugation (2500 rpm for 5 minutes) after the addition of ethyl ether (~3 ml); sediments were examined for the presence of small trematode eggs [10,15]. Positive and negative individuals were divided by gender, age group, and community; univariate associations between these variables and small trematode egg infection status (positive/negative) were addressed using chi-square tests with a criterion of statistical significance of *p* = 0.05.

### Morphological characteristics of opisthorchiid eggs

Eggs from the families Opisthorchiidae and Heterophyidae measuring <30 μm in length have been labeled as small trematode eggs because their morphological similarities prevent detailed discrimination via light microscopic diagnosis [17–19]. Human cases were tagged as positive based on the presence of at least one egg with the following characteristics: pyriform with a length of 20–30 μm, operculated with ‘shoulders’, and a ‘knob’ on its abopercular end [13,17,18]. Other parasite species whose eggs were found in feces will be reported elsewhere.

### Recovery and observation of adult worms

One liver of a cat and one from a dog were examined for liver flukes, both animals coming from the communities studied. According to the owners, the cat had died of an unknown disease, and the dog had drowned; we obtained owners' permission to obtain samples from the deceased animals. Livers were immersed in saline solution, sliced in sections to identify bile ducts, and compressed. One entire family of egg-positive human cases agreed voluntarily to be treated with praziquantel [13] and purged with a dose of sodium picosulfate (5 mg single dose) to collect diarrheic stools. We filtered the mixed stools of the entire family with a mesh of 2 mm and after three cleaning rounds with water after sedimentation, we examined the remains with the dissection microscope [30,31]. Morphological identification of adult worms was performed by fixating adults with 70% alcohol after compression between two glass slides; these samples were stained with borax carmine, and mounted with Canada balsam [10].

### Collection and identification of fish

Freshwater fish were purchased from local residents of each community; they collect fish using casting nets, and eat them as a source of protein. Fishes were divided by morphology, and identified at species level using publicly available taxonomic keys of Ecuadorian freshwater fish [32,33] with expert confirmation. From the fish collected, we removed scales, fins, viscera, and heads, and used only muscle tissue to search for metacercariae, since the same procedure is applied when local people prepare fish for consumption. In the same way, we processed fish identified by the local populations as edibles, and discarded those identified as non-edible (e.g., *Astroblepus* spp.). Fish were transported to the laboratory facilities in Quito in cooler cages at 4°C.

### Collection of metacercariae through fish artificial digestion

Fish of the same species were digested artificially to recover live metacercariae [10,34,35]. Briefly, after removing scales, fin, viscera, and head from each group of fish, all of the remaining tissue was mixed with 200 ml of artificial digestive solution (hydrochloric acid 3.5% + pepsin 0.5%) and 800 ml of warm water (37°C). This solution was left to rest for approximately 1 hour in a shake temperature incubator. The digestive solution was cleared up by passing the contents through three sieves, ordered by mesh diameter from 500, 300, and 105 μm. The contents of the last sieve were reversed and washed to recover contents between 105 and 300 μm [10,34,35]. We examined this sediment with a dissecting microscope, and isolated metacercariae with morphological features of opisthorchiid trematodes using published descriptions [10,25,35,36]. We used an optical microscope to explore further the morphology of different metacercariae, and proceeded to perform molecular identification.

### Morphological and molecular identification of metacercariae

Given the morphological similarities of metacercariae of the Opisthorchiidae and Heterophyidae [10,35] and the lack of studies of liver or intestinal fish-borne trematodes in Ecuador, we only analyzed metacercariae with appropriate morphology (see above). We followed a similar approach as described by Calvopiña et al. 2018 [37], including DNA extraction, polymerase chain reaction (PCR), PCR-restriction fragment length polymorphism (RFLP), and DNA sequencing. In brief, we extracted DNA from individual metacercariae using the QIAamp DNA Micro Kit (Qiagen, Stanford, California, United States). We amplified the internal transcribed spacer 2 (ITS2) ribosomal RNA gene using conserved inter-species primers for trematodes: the 3S forward (3S-F) primer, a product of 26 base pairs (bps; 5’-GGTACCGGTGGATCACTCGGCTCGTG-3’ [38]) and the A28 reverse (A28-R) primer, a product of 29 bps (5’- GGGATCCTGGTTAGTTTCTTTTCCTCCGC-3’[39]). PCR amplification was performed in a thermal cycler (TaKaRa PCR Thermal Cycler Dice Gradient, Takara Bio, Shiga, Japan) with the DNA template (50 ng in 0.05 mL) and 2.5 U Phusion High-Fidelity DNA Polymerase (Thermo Fisher Scientific, Waltham, Massachusetts, United States) as follows: (1) DNA denaturation at 98°C for 30 seconds, (2) 30 cycles of denaturation at 98°C for 10 seconds, annealiation at 55°C for 10 seconds, and extension at 72°C for 15 seconds; (3) the final extension was set at 72°C for 7 minutes. For PCR-RFLP, we used the restriction enzyme *Hind* III (New England Biolabs, Ipswich, Massachusetts, United States), which targets a specific region of ITS2. The enzyme digestion was performed using 10 μl of PCR amplicons with 5 U of *Hind* III for one hour at 37°C. Digested/undigested PCR products were run in electrophoresis agarose gels (2% w/v) to discriminate fragments; we included DNA from adult *Amphimerus* sp. from Esmeraldas [13] as positive control. Finally, DNA sequences from some of the amplified products were obtained using BigDye Terminator v3.1 Cycle Sequencing Kit and the automatic sequencer 3730xl DNA Analyzer (Thermo Fisher Scientific) with the aforementioned primers. We analyzed DNA sequences with GENETYX-Win software (Genetyx Co., ver. 13, Tokyo, Japan) using the nucleotide database from the National Center for Biotechnology Information (NCBI) and with *Amphimerus* sp. sequences (GenBank accession Nos.: AB678442 and AB926430).

## Results

### Human samples

We collected and analyzed 176 fecal samples from individuals of the three communities. Small trematode eggs were found in 63/176 (35.8%) samples. Among positives individuals, age ranged from 1 to 83 years (average = 31.6, standard deviation (SD) = 22.9), with a female/male ratio of 1:1.04 (31 males and 32 positive females). No statistical association existed between age or gender and presence of small trematode eggs (Table 1). No statistical associations were found between communities and positive cases; community C was the one with the largest population delivering fecal samples and positive cases (31/91, 34.1%), although community B had a higher prevalence (25/61, 41%; Table 1 and Fig 1).

**Table 1:** Human population showing small trematode eggs in Pedro Pablo Gómez communities. Participants were divided by age, gender, and communities to assess associations with small trematode egg presence, although none was statistically significant. Percentages are calculated with the totals of each row.

Age (years)	Positive (%)	Negative	Total (n)	Chi-square test	Degrees of freedom	*p* value
1–19	29 (39.2)	45	74	2.67	3	0.45
20–39	10 (25)	30	40			
40–59	12 (37.5)	20	32			
≥60	12 (40)	18	30			
Total cases	63 (35.8)	113	176			
Gender						
Females	32 (37.2)	54	86	0.15	1	0.7
Males	31 (34.4)	59	90			
Total cases	63 (35.8)	113	176			
Community						
A	7 (29.2)	17	24	1.29	2	0.52
B	25 (41)	36	61			
C	31 (34.1)	60	91			
Total cases	63 (35.8)	113	176			

### Morphological and molecular identification of adult specimens

**Fig 2:**
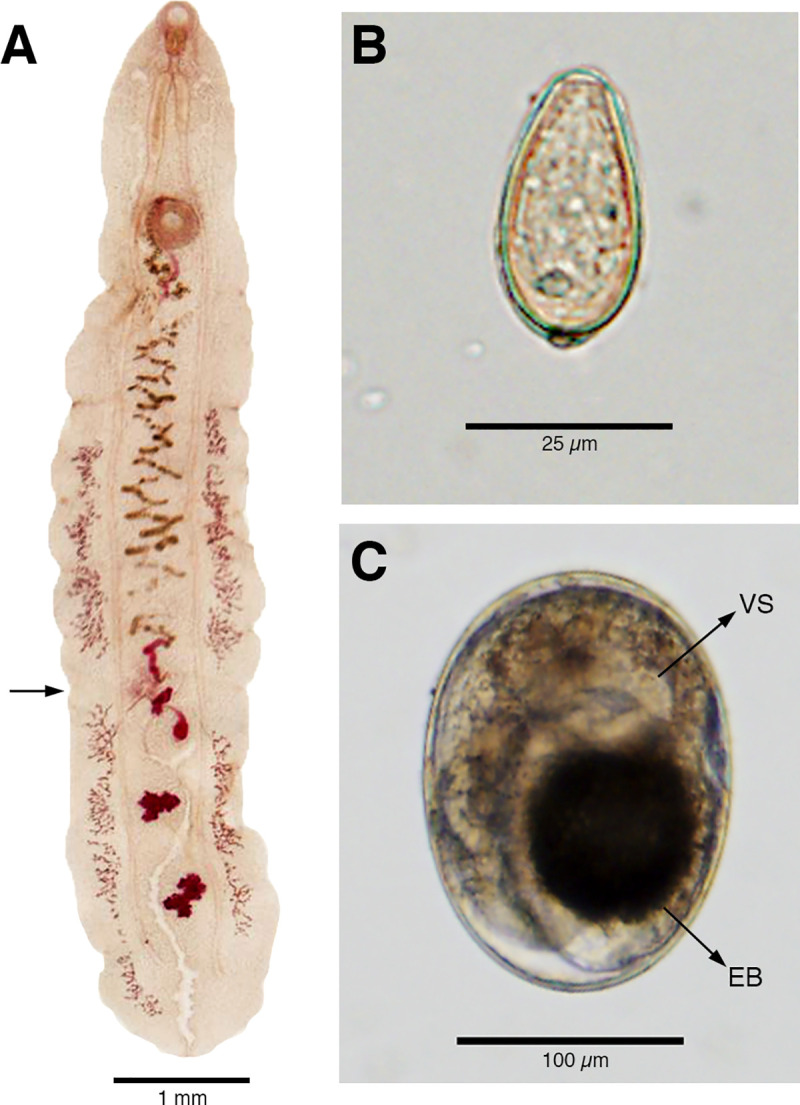
Characteristics of *Amphimerus* sp. in different morphological stages from Pedro Pablo Gómez, Manabí, Ecuador. (A) Adult *Amphimerus* sp. fluke isolated from the liver of a naturally infected dog. Note the anterior-posterior division of the ungrouped vitelline glands (arrow) at the level of the ovary, which extend beyond the testes reaching the posterior end of the body. (B) Egg isolated from a human fecal sample resembling characteristic opisthorchiid and heterophyid eggs: pyriform shape, anterior operculum, and an apparent posterior spine (knob). (C) Metacercaria of *Amphimerus* sp. isolated from fish collected in the communities of Pedro Pablo Gómez, showing an oval shape within a cyst wall; the larva inside features a round and prominent ventral sucker (VS) and a black, round to oval, excretory bladder (EB).

We isolated 18 and 45 adult flukes from biliary ducts of the dog and cat livers respectively. In all cases, worms of ~10 mm length had bilateral, ungrouped vitelline glands, divided at the level of the ovaries into anterior and posterior sections. Vitelline glands were distributed beyond the round, partially lobed testes, reaching the posterior end of the adult fluke body (Fig 2). From the seven-member human family, we found three fragmented opistorchiid-like adult trematodes through our praziquantel plus purging approach. Our PCR-RFLP protocol split the DNA of all the adult fluke samples in two fragments, as those expected for *Amphimerus* sp. (see below). Sequences of amplified digested products aligned with those previously identified as *Amphimerus* sp. in Ecuador (GenBank accession Nos.: LC490158-161). We did not find other trematodes in human stools or the intestines of the dog and cat.

### Fish species and identification of metacercariae

**Fig 3:**
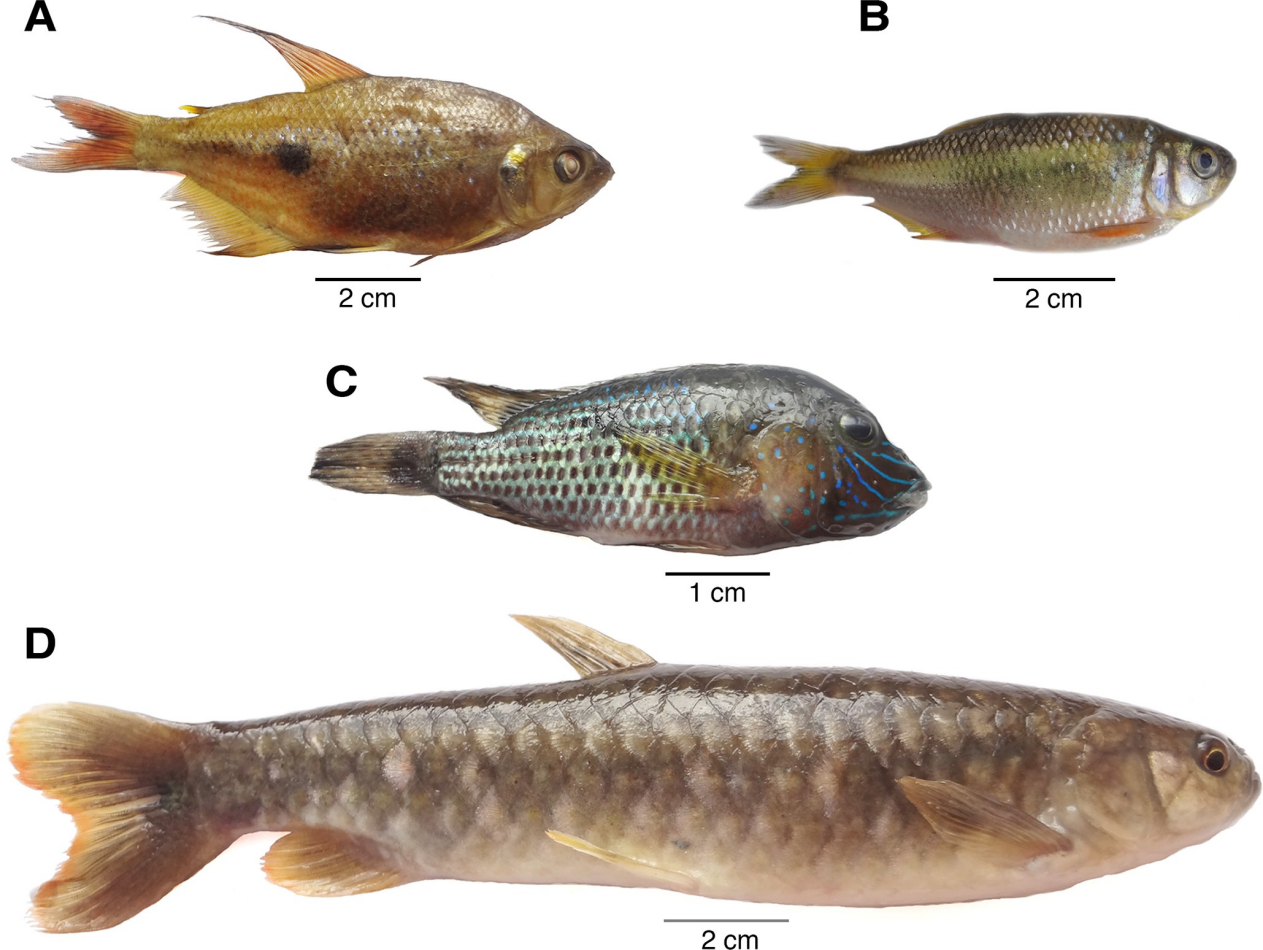
Freshwater fish incriminated as potential second intermediate hosts of *Amphimerus* sp., collected in Pedro Pablo Gómez, Manabí, Ecuador. All fish species were positive for *Amphimerus* sp. metacercariae. (A) *Rhoadsia altipinna*, known locally as ‘ancha’. (B) *Bryconamericus bucay*, known locally as ‘engorda’. (C) *Andinoacara rivulatus*, known locally as ‘vieja’. (D) *Piabucina aureoguttata*, known locally as ‘guaija’. Pictures by Daniel Romero-Alvarez.

**Fig 4:**
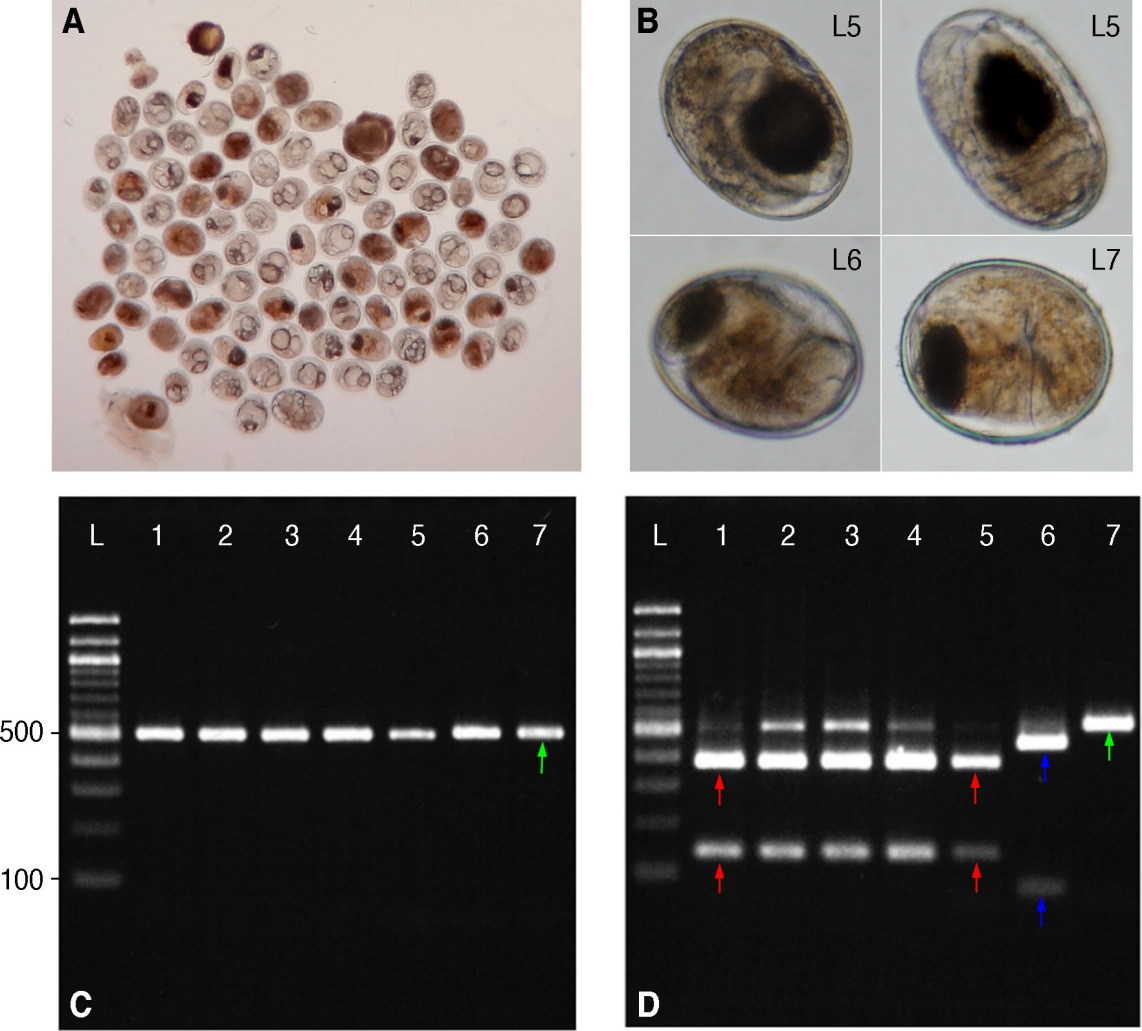
Molecular diagnosis of *Amphimerus* sp. metacercariae based on PCR-RFLP. (A) Multiple metacercariae were isolated from fish collected at each community. (B) Different metacercariae identified based on PCR-RFLP as shown in panel D. (C) First round of PCR amplification using universal trematode primers (green arrow). (D) Digested (L1-L6) and undigested (L7) PCR products by the restriction enzyme *Hind* III. Lanes 1–5 corresponds to (L1) adult *Amphimerus* sp. flukes from a human case at Esmeraldas, followed by adult parasites from a human (L2), a dog (L3), and a cat (L4), and (L5) *Amphimerus* sp. metacercariae from freshwater fish (i.e., *Bryconamericus bucay*) from Pedro Pablo Gómez; the two fragments correspond to 374 and 140 base pairs (red arrows). Lane 6 corresponds to the metacercariae of *Haplorchis pumilio* with fragments of 440 and 86 base pairs (blue arrows). Lane 7 corresponds to unidentified metacercariae from fish in Pedro Pablo Gómez. L: 100 base pairs DNA Ladder (New England Biolabs, Ipswich, Massachusetts, United States).

We collected 649 fish belonging to four species: *Rhoadsia altipinna* (n = 504, 77.66%, family = Characidae, locally known as ‘ancha’), *Bryconamericus bucay* (n = 80, 12.33%, family = Characidae, locally known as ‘engorda’), *Andinoacara rivulatus* (n = 58, 8.93%, family = Cichlidae, locally known as ‘vieja’), and *Piabucina aureoguttata* (n = 7, 1.08%, family = Lebiasinidae, locally known as ‘guaija’, Fig 3). Through artificial digestion experiments, we found numerous metacercaria in all of the fish species (Figs 3 and 4).

In total, we separated 101 individual metacercariae for molecular diagnosis from the fish species examined belonging to each of the three communities (Table 2). Molecular amplification with PCR yielded a product of ~500 bps (Fig 4). The restriction enzyme *Hind* III digested some PCR amplicons in two fragments, with 374 (i.e., 3S-F: 26 + 5’-ITS2 section: 348 = 374 bps) and 140 bps (i.e., 3’-ITS2 section: 111 + A28-R: 29 = 140 bps) following the pattern of adult flukes of *Amphimerus* sp. isolated in Esmeraldas and those isolated in PPG (Fig 4). We found 34 of 101 (33.6%) metacercariae matching the fragment sizes of *Amphimerus* sp. by this method (Fig 4). We sequenced ten of these samples obtaining DNA identical to that of the adult *Amphimerus* sp. isolated from the dog and the cat in this study (GenBank accession Nos.: LC490160-LC490162) with two nucleotide differences with sequences previously published [13,14], and the adult worm recovered from humans (GenBank accession No.: LC490158). From the other amplicons, 29/101 (28.7%) were also digested by *Hind* III and divided into two fragments with different sizes from those of *Amphimerus* sp. (Fig 4), namely, 86 (i.e., 3S-F: 26 + 5’-ITS2 section: 60 = 86 bps) and 440 bps (i.e., 3’-ITS2 section: 411 + A28-R: 29 = 440 bps); five of these products were sequenced and identified as *Haplorchis pumilio*, with a 100% match with one of the available GenBank sequences (Accession No.: JX532163, [40]). The RFLP primer used in this study was unable to digest any of the remaining 38/101 (37.6%) PCR products (Fig 4 and Table 2). By sequencing, we were able to identify only *Pygidiopsis genata* (Community A, *A*. *rivulatus* n = 5, *R*. *altipinna* n = 1; 99.56% similarity with GenBank sequence No.: AY245710.1 [41]) and *Centrocestus formosanus* (Community B, *A rivulatus* n = 1; 99.79% similarity with GenBank sequence No.: KY075665.1), trematodes of the Heterophyidae family.

**Table 2:** Metacercariae identified by molecular diagnosis. Metacercariae isolated from fish of the three communities studied were tested by PCR-RFLP and DNA sequencing to identify trematode species in the area. Percentages are calculated with the totals of each row.

Fish species	*Amphimerus sp*.	*Haplorchis pumilio*	Others	Total
*Rhoadsia altipinna*	10 (29.4%)	11 (32.4%)	13 (38.2%)*	34
*Bryconamericus bucay*	10 (37%)	6 (22.2%)	11 (40.7%)	27
*Andinoacara rivulatus*	2 (7.4%)	11 (40.7%)	14 (51.9%)**	27
*Piabucina aureoguttata*†	12 (92.3%)	1 (7.7%)	0 (0%)	13
Total	34 (33.6%)	29 (28.7%)	38 (37.6%)	101 (100%)

†: Fish species were obtained from all the communities except for *P*. *aureoguttata* in community B.

*/**: Other trematodes identified by sequencing include: *Pygidiopsis genata (n = 6) and **Centrocestus formosanus (n = 1).

From examination of the metacercariae identified molecularly as *Amphimerus* sp., we propose the following characteristics as guide for identification: an encysted, oval-shaped metacercaria with a mean size of 179.9 μm (range = 155.5–205.5 μm, SD = 14, n = 20) by 125.4 μm (range = 102.7–138.8 μm, SD = 9.4, n = 20); a round, prominent, and easily identifiable ventral sucker (diameter mean = 48.8 μm, SD = 3.9, n = 14); and an oval to round dark excretory bladder (Fig 2). These features resemble morphologically the metacercariae of *A*. *elongatus*, as described by Font (1991) and Wallace (1939) [25,36]. For comparison, we measured the diameter of the *H*. *pumilio* metacercariae sequenced, finding a mean size of 181.8 μm (range = 188.9–175 μm, SD = 5, n = 5) by 140.3 μm (range = 133.3–150, SD = 6.2, n = 5; thus, less elliptical), without a visible ventral sucker (Fig 4). Metacercariae from other trematodes followed a measurement closer to that of *H*. *pumilio* (mean = 180.4 μm, range = 169.4–208.3 μm, SD = 19.9 by 143.2 μm, range = 125–163.9 μm, SD = 10.5, n = 13) with an either apparent or unapparent ventral sucker (Fig 4).

## Discussion

**Fig 5:**
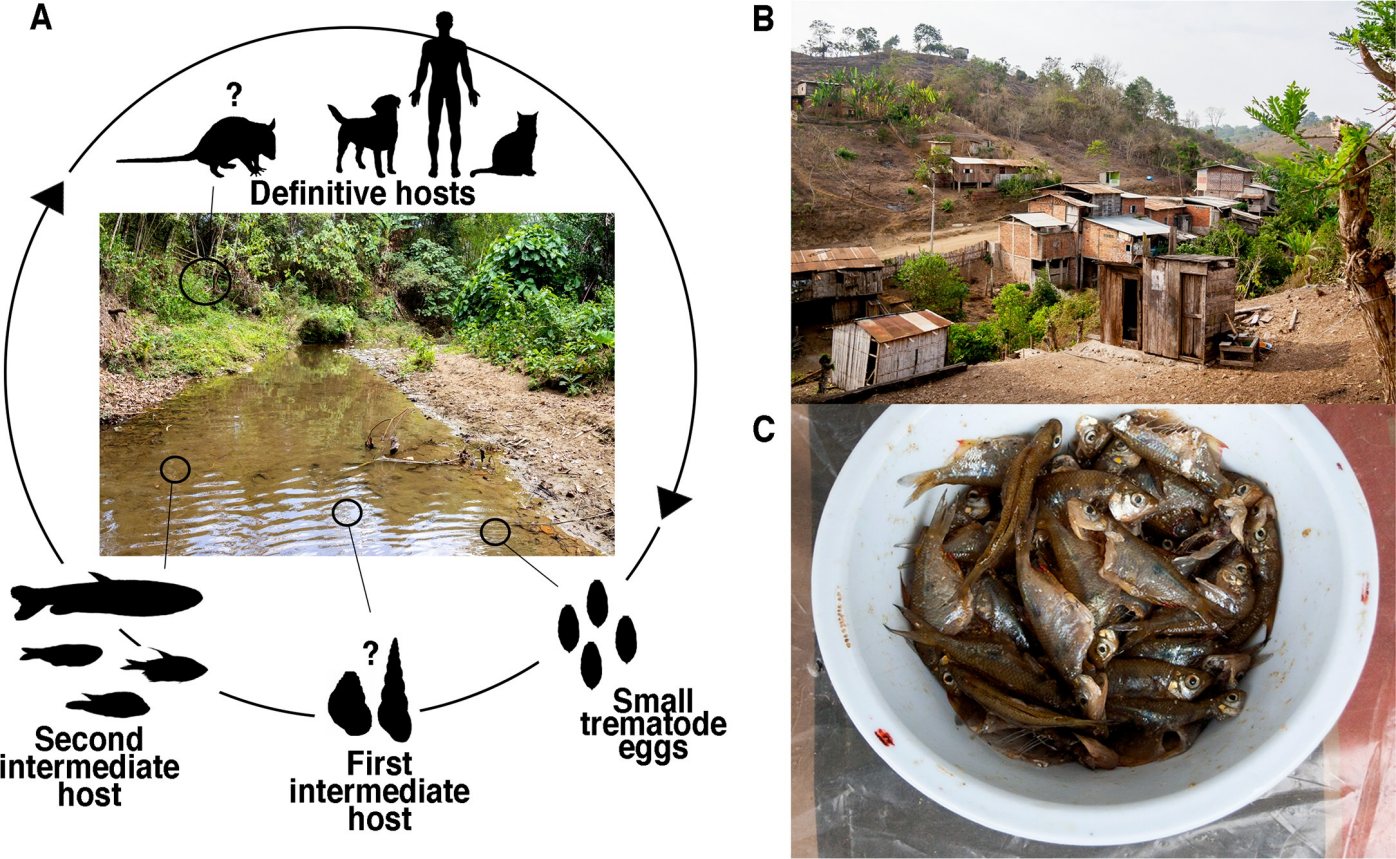
Schematic life cycle of *Amphimerus* sp. in Manabí province, Ecuador. (A) All opisthorchiids use two intermediate hosts (prosobranch mollusc and fish for first and second, respectively); we lack information on the snail species involved in *Amphimerus* sp. life cycle in Ecuador. Opossums play a role in the sylvatic maintenance of *A*. *neotropicalis* and *A*. *pseudofelineus* in Latin America [42] and might be incriminated in the life cycle of *Amphimerus* sp. in Ecuador as well. (B) Landscape of rural community B within Pedro Pablo Gómez (PPG) parish, Manabí province, Ecuador. (C) Local dish being prepared in one of PPG studied communities from fish collected in surrounding streams.

The studied communities of Manabí province in Ecuador have been incriminated as liver-fluke endemic since 1949 by the detection of small trematode eggs in humans, and eggs and adult flukes in dogs [11]. Although first described as *O*. *guayaquilensis*, posterior taxonomic classifications agreed that the specimens belonged to the genus *Amphimerus* [42,43] and therefore pioneered the first description of the parasite in Ecuador. In the present study, we show that the area remains highly endemic for amphimeriasis and potentially other flukes. First, we have identified molecularly adult *Amphimerus* sp. from feces of humans and bile ducts of naturally infected domestic animals, whose DNA sequences match those of *Amphimerus* sp. from the province of Esmeraldas, ~325 km north of the present study sites (Figs 1 and 4; [13]). Second, we have isolated and identified metacercariae of *Amphimerus* sp. in four edible freshwater fish species collected in the area (i.e., *R*. *altipinna*, *B*. *bucay*, *A*. *rivulatus*, and *P*. *aureoguttata*; Fig 3 and Table 2), incriminating for the first time second intermediate hosts for *Amphimerus* sp. Third, we have reported the presence of small trematode eggs in fecal samples from humans at an overall prevalence of 35.8% (Figs 2 and 5 and Table 1). Finally, although beyond our primary goals, we consistently found the presence of *H*. *pumilio* metacercariae in the same edible fishes suggesting the possibility of human infection by this heterophyid intestinal fluke (Table 2).

The endemic area of amphimeriasis in the riverine communities alongside the Cayapas River at the province of Esmeraldas (Fig 1) differs from PPG both ecologically and epidemiologically. The Cayapas River area belongs to the Chocó region, from the tropical and subtropical moist broadleaf forest biome [28]; roughly, it has a mean annual temperature of ~23–26°C and a mean annual precipitation of ~2000 mm [44]. A mean prevalence of 23.9% (71/297) of small trematode eggs was reported in humans among three villages in 2011, whereas a prevalence of 36.2% (38/105) was reported recently (2018) in a more remote village, not included in the previous study [15]; access to these villages depends mainly on boats. These rural populations are composed of indigenous native Ecuadorians of the ‘Chachi’ ethnicity who catch freshwater fish, and prepare and eat smoked fish dishes [13]. In contrast, the high prevalence of 35.8% in the present study corresponds to a population of mestizo inhabitants [29] who instead of being connected by rivers, live in a human-populated, dry forest landscape connected by roads, and watered by small streams feeding into the Pedro Pablo Gómez and Grande Rivers (Figs 1 and 5); in this area, use of fish as a daily food source is secondary. Primary protein sources include seafood and livestock products; however, when freshwater fish are eaten, they are prepared as raw fish with salt and lemon juice (Fig 5). Consumption of infected fish to sustain or complement diets for the populations in Esmeraldas and Manabí, respectively, is an ideal means of maintaining the life cycle of *Amphimerus* sp. and potentially other trematodes [13,45] (Fig 5).

We found metacercariae of *Amphimerus* sp. in all fish species recognized by local people as edible, belonging to three different families (i.e., Characidae, Cichlidae, and Lebiasinidae, Fig 3 and Table 2). Considering the high number of fish species involved in the life cycles of other opistorchiid trematodes, such as *C*. *sinensis* and *O*. *viverrini* (32 fish species [46] and 11 fish species, respectively [47]), we surmise that our number is an underestimation of the total number of fish species involved in the life cycle of *Amphimerus* sp.; regardless, a limited number of second intermediate hosts has been reported for the genus elsewhere [9,25].

In the present study, we did not quantify fish infection prevalence or intensity of infection, which is an important limitation; we considered that addressing this point with our current sampling could bias our results. For instance, *P*. *aureoguttata* is a fish that spends its time on river bottoms, and is therefore missed if fish are captured with casting nets (e.g., no samples of this species were collected in community B, Table 2). On the other hand, *R*. *altipinna* and *B*. *bucay* are easily captured by casting nets considering their behavior [32]. Therefore, for the present study, presence of metacercariae in these fish incriminate them as second intermediate hosts of *Amphimerus* sp. and open possibilities for pragmatic preventive measures.

Although the three characteristics suggested for the identification of *Amphimerus* sp. metacercariae might be helpful to identify it for future studies, we stress that almost the same characteristics could also be present in metacercariae of *H*. *pumilio* or some other species (Figs 2 and 4 and Table 2 [10,30,35]). Because we can barely rely on morphology to distinguish eggs or metacercariae from members of the Opisthorchiidae and Heterophyidae families, we recommend to implement the PCR-RFLP molecular approach presented in this study in epidemiological surveys aiming to identify fish species involved in *Amphimerus* sp. life cycle (Fig 5) and to survey others regions of Ecuador where consumption of undercooked freshwater fish is frequent in order to effectively discriminate its metacercariae in a laboratory context (Fig 4).

As mentioned earlier, Rodríguez et al. reported the causative agent of liver-fluke infections in humans and dogs in the area as *O*. *guayaquilensis* in 1949 [11]. However, the lack of consensus on *Opisthorchis* spp. geographic distributions at that time, and on the taxonomy classification of the parasite, led researchers to consider it first a synonym of *A*. *pseudofelineus* [43], and later as a distinct species (*A*. *guayaquilensis*) [42]. The evidence collected in the present study allows us to identify species to genus: namely, the adult parasite has vitelline glands divided in anterior and posterior sections at the level of the ovary, a key taxonomic feature for the genus *Amphimerus* (Fig 2). Our findings highlight the similarities between the adult parasites described here with those described previously [11,13]; *Amphimerus* sp. adult flukes had vitelline glands extending beyond the testes (Fig 2), which potentially could be a morphological feature for identification of the species [10,42]. Although morphologically and molecularly (i.e., PCR-RFLP) *Amphimerus* sp. from Esmeraldas and Manabí provinces are identical, we found a difference of two nucleotides within the ITS2 sequence examined (GenBank sequences: AB678442 and AB926430 vs. LC490160 and. LC490162). We suggest that a two nucleotide difference in the 459 bps sequence section of ITS2 might be a signal of intraspecific variability that should be examined in detail; therefore, we assert that the species status of this parasite is still unresolved and needs further investigation using sensitive molecular and genomic tools [9,10,14,42,48].

The identification of opisthorchiid-like eggs in humans in Esmeraldas has been acknowledged as liver-fluke infections due to *Amphimerus* sp. [13,14], however, we evidenced in this study a more complex situation potentially involving multiple intestinal-fluke species of Heterophyidae such as *H*. *pumilio* identified in the four fish species and throughout the studied communities (Fig 4 and Table 2). Liver and intestinal-fluke infection should be considered in differential diagnosis of small trematode eggs detected in Ecuador [49,50]. Besides *H*. *pumilio*, we also identified the heterophyids *C*. *formosanus* in *A*. *rivulatus* of community B, and *P*. *genata* in *R*. *altipinna* and *A*. *rivulatus* in community A (Table 2). Both intestinal-flukes are largely understudied in South America and agreement on its distribution is still being revised [50,51]. In fact, our discovery here was incidental and therefore we remain cautious on their status in Ecuador. For instance, *C*. *formosanus* dwells mainly in the gills of fish while *P*. *genata* usually dwells in the muscle and other organs [52,53]. In this study, we discarded all fish structures except for the muscle since we aimed to identify *Amphimerus* sp. metacercariae, which we expected to be found encysted there [10,35]. Further studies should consider these preliminary detections to focus on clarify the richness and abundance of trematodes as living species—besides only as disease agents—; for these investigations, the entire fish should be examined with the artificial digestion technique.

Pulido-Murillo et al. (2018) recently found evidence of *H*. *pumilio* and *C*. *formosanus* in the snail *Melanoides tuberculata* in the department of Lima, Peru [45]. The invasive snail *M*. *tuberculata* was found throughout our study area, sharing ponds and streams with the fish species identified as positive here; however, we did not attempt to search for cercariae or test potential *Amphimerus* sp. first intermediate hosts (Fig 5). *P*. *genata* also uses *M*. *tuberculata* as first intermediate host [49]; research of this invasive snail might help understand the distribution of these and other metacercariae unidentified in our study.

The high prevalence of small trematode eggs in the human population of the three communities reported here may still be an underestimate considering the low sensitivity of small trematode egg detection using the formalin-ether concentration technique and one-time fecal sample examination, as we have shown recently [15]. The Kato-Katz coprological technique, immunological, or LAMP-based methods of diagnosis should be encouraged to characterize the true prevalence of human-fluke infections in current endemic areas, discover new foci of human infections, and to complete the knowledge of other components of the *Amphimerus* sp. life cycle (e.g., definitive wildlife hosts or snails) [21–23,54].

Understanding the life cycle of zoonotic helminths is paramount to guiding interventions. Recognition of these local fish as a source of metacercariae of liver (i.e., *Amphimerus* sp.) and intestinal flukes (e.g., *H*. *pumilio*) could prompt development of public health education campaigns regarding safe food practices and sanitation, with multisectorial involvement including research groups, community members, and relevant government sectors for effective monitoring, surveillance, and control [2,16,55]. For example, interventions in Thailand effectively reduced the prevalence of *O*. *viverrini* infections among the communities of Khon Kaen Province by putting together, among others, human chemotherapy with praziquantel, installation of latrines, and health education targeting behavioral changes on fish intake [56]; these three objectives could also be used to interrupt the transmission of *Amphimerus* sp. in Ecuador. Open research questions regarding this system include (1) determination of whether infections with *Amphimerus* sp. play a role in development of clinical manifestations in human cases (see [57]), (2) identification of primary intermediate hosts in PPG, and first and second intermediate hosts in the Cayapas River area, (3) clarification of the taxonomic status of the local *Amphimerus* populations, and (4) documentation of the potential geographic ranges of liver fluke infections in Ecuador and other American countries.

## Supporting information

S1 File Strengthening the Reporting of Observational Studies in Epidemiology (STROBE) statement. (PDF)
