# Supplementary material for: Liver fluke infections by Amphimerus sp. (Digenea: Opisthorchiidae) in definitive and fish intermediate hosts in Manabí province, Ecuador
Source: PLoS Negl Trop Dis. 2020 Jun 29;14(6):e0008286. doi: 10.1371/journal.pntd.0008286 (PMC7351216; doi:10.1371/journal.pntd.0008286)
Supplement: S1 File — (PDF) [file pntd.0008286.s001.pdf]

S1 File. Strengthening the Reporting of Observational Studies in Epidemiology (STROBE) statement. Checklist filled according to the available items for cross-sectional studies download from https://www.strobe-statement.org/index.php?id=available-checklists.

STROBE Statement—Checklist of items that should be included in reports of *cross-sectional studies*

	Item No	Recommendation
**Title and abstract**	1	(*a*) Indicate the study's design with a commonly used term in the title or the abstract. **See Methods/Principal Findings in Abstract page 2; Material and Methods, paragraph 1, page 7.**
		(*b*) Provide in the abstract an informative and balanced summary of what was done and what was found. **See Abstract, pages 2-3.**
**Introduction**		
Background/rationale	2	Explain the scientific background and rationale for the investigation being reported. **See Introduction, pages 5-7.**
Objectives	3	State specific objectives, including any prespecified hypotheses. **See Introduction, paragraph 5, page 7.**
**Methods**		
Study design	4	Present key elements of study design early in the paper. **See Methods, paragraph 1-4, pages 7-10.**
Setting	5	Describe the setting, locations, and relevant dates, including periods of recruitment, exposure, follow-up, and data collection **See Methods, paragraph 1, page 8. See also Fig 1.**
Participants	6	(*a*) Give the eligibility criteria, and the sources and methods of selection of participants **See Methods, paragraph 2, page 9.**
Variables	7	Clearly define all outcomes, exposures, predictors, potential confounders, and effect modifiers. Give diagnostic criteria, if applicable **We are describing presence/absence of small trematode eggs in humans associated with age groups, gender, and community studied (See Methods, paragraph 2-3, page 9-10). Human diagnostic criterion is available in Methods, paragraph 3, page 9-10. We are assessing *Amphimerus* spp. adults and metacercariae presence/absence in humans, a dog and a cat (Methods, paragraph 4, page 10), and fish (Methods, paragraph 5, page 11), respectively without establishing other correlation/causality relationships.**
Data sources/ measurement	8*	For each variable of interest, give sources of data and details of methods of assessment (measurement). Describe comparability of assessment methods if there is more than one group **For parasitological approaches see Methods paragraphs 2-6, pages 7-11; for molecular approaches see Methods paragraph 7, pages 12-13.**
Bias	9	Describe any efforts to address potential sources of bias **As a descriptive research, we are explicitly declaring the sources of bias on the study design: see Discussion, paragraphs 4-7, pages 26-28.**
Study size	10	Explain how the study size was arrived at **As a descriptive research we did not calculate any particular sample size and**

		**limited our approach to the availability of collected material. See Methods section, paragraph 2, pages 9.**
Quantitative variables	11	Explain how quantitative variables were handled in the analyses. If applicable, describe which groupings were chosen and why<br>**Patients were divided in positive/negative according to the presence/absence of small trematode eggs. See Methods, paragraph 2-3, pages 9-10.**
Statistical methods	12	(*a*) Describe all statistical methods, including those used to control for confounding<br>**We used a Chi-squared test to explore associations between age groups, gender, and community, and small trematode eggs positivity. Due to the lack of statistical significance we did not perform any further statistical test. See Methods, paragraph 2, pages 9-10.**
		(*b*) Describe any methods used to examine subgroups and interactions<br>**Not applicable.**
		(*c*) Explain how missing data were addressed<br>**We included all the data available.**
		(*d*) If applicable, describe analytical methods taking account of sampling strategy<br>**Not applicable.**
		(*e*) Describe any sensitivity analyses<br>**Not applicable.**
**Results**		
Participants	13*	(a) Report numbers of individuals at each stage of study—eg numbers potentially eligible, examined for eligibility, confirmed eligible, included in the study, completing follow-up, and analysed<br>**See Results, paragraph 1-4, pages 14-20.**
		(b) Give reasons for non-participation at each stage<br>**Not applicable.**
		(c) Consider use of a flow diagram<br>**Not applicable.**
Descriptive data	14*	(a) Give characteristics of study participants (eg demographic, clinical, social) and information on exposures and potential confounders<br>**See Results, paragraph 1, pages 14-15.**
		(b) Indicate number of participants with missing data for each variable of interest<br>**Not applicable.**
Outcome data	15*	Report numbers of outcome events or summary measures<br>**See Tables 1-2.**
Main results	16	(*a*) Give unadjusted estimates and, if applicable, confounder-adjusted estimates and their precision (eg, 95% confidence interval). Make clear which confounders were adjusted for and why they were included<br>**Not applicable.**
		(*b*) Report category boundaries when continuous variables were categorized<br>**See Results, paragraph 1, pages 14-15, also Discussion paragraph 3-4, pages 25-26.**
		(*c*) If relevant, consider translating estimates of relative risk into absolute risk for a meaningful time period<br>**Not applicable.**
Other analyses	17	Report other analyses done—eg analyses of subgroups and interactions, and sensitivity analyses

		**Qualitative molecular analysis performed to detect presence/absence of the parasites studied are described in Methods, paragraph 7, pages 12-13 and Results, paragraph 4, pages 19-20.**
**Discussion**		
Key results	18	Summarise key results with reference to study objectives **See Discussion, paragraph 1, page 22-23.**
Limitations	19	Discuss limitations of the study, taking into account sources of potential bias or imprecision. Discuss both direction and magnitude of any potential bias. **As a descriptive research, we are explicitly declaring the sources of bias on the study: see Discussion, paragraphs 3-6, pages 25-28.**
Interpretation	20	Give a cautious overall interpretation of results considering objectives, limitations, multiplicity of analyses, results from similar studies, and other relevant evidence **See Discussion paragraph 2-6, pages 24-29.**
Generalisability	21	Discuss the generalisability (external validity) of the study results **See Discussion, paragraph 2-6, pages 24-29.**
**Other information**		
Funding	22	Give the source of funding and the role of the funders for the present study and, if applicable, for the original study on which the present article is based **See corresponding subsection at PLoS NTD submission system.**

*Give information separately for exposed and unexposed groups.

Note: An Explanation and Elaboration article discusses each checklist item and gives methodological background and published examples of transparent reporting. The STROBE checklist is best used in conjunction with this article (freely available on the Web sites of PLoS Medicine at http://www.plosmedicine.org/, Annals of Internal Medicine at http://www.annals.org/, and Epidemiology at http://www.epidem.com/). Information on the STROBE Initiative is available at www.strobe-statement.org.
